# The influence of the digital divide on emergency remote student-centred learning during the COVID-19 pandemic: a case study of journalism education

**DOI:** 10.1007/s43545-023-00626-6

**Published:** 2023-02-20

**Authors:** Sisanda Nkoala, Trust Matsilele

**Affiliations:** Media Department, Cape Peninsula University of Technology, 80 Roeland Street, Cape Town, 8001 South Africa

**Keywords:** Digital divide, Usage gap, Student-centred learning, Journalism education, COVID-19

## Abstract

This paper explores the emergency remote learning experiences of journalism students. It discusses how disparities in access to digital tools and participation in online learning, caused by the digital divide, influenced how some benefitted from the student-centred learning approaches adopted while others could not. The study seeks to answer this question: To what extent did the digital divide influence the experiences of journalism students with emergency remote student-centred learning adopted due to the 2020 COVID-19 pandemic? The study uses Van Dijk’s theory of the usage gap to argue that the unequal access to digital technologies experienced by some students brought about unequal participation in learning. This is despite the use of more student-centred approaches, which, according to existing literature, are supposed to foster greater engagement and participation. The data consisted of 113 vlogs created by second and third-year students from the Cape Peninsula University of Technology in Cape Town, South Africa, between June 1, 2020, and June 30, 2020.

## Introduction

The 2020 COVID-19 pandemic fundamentally shifted practices across fields, disciplines and professions. Perreault and Perreault (2021) argue that for the field of journalism, the pandemic pushed practitioners and scholars to be more reflective. Because journalism education is reflexive and closely related to industry, these changes affected journalism studies. Fowler-Watt et al. (2020) argue that the pandemic presented a unique challenge for journalism educators striving to teach professional values online. It disrupted the normative ways of conducting journalism studies and, to a degree, in contexts like South Africa, mimicked the apartheid-style of a “separate but equal” system (Matsilele and Nkoala 2022). This challenge was also compounded by the pandemic sharpening the existing inequalities within the higher education space. For example, historically disadvantaged institutions struggled  to supply their students with adequate digital tools to allow uninterrupted learning compared to traditionally white institutions. These challenges posed an existential threat to journalism education, especially in terms of producing under-equipped graduates, as some were not able to participate meaningfully in critical elements of their studies. This, in turn, imposed a need to reconfigure education in this field. While the fundamentals of what it means to be a journalist have not changed, the craft has evolved quite notably (Posetti et al. 2020), which has changed approaches to journalism education.

This paper explores the experiences of journalism students with the emergency remote education necessitated by the COVID-19 pandemic. It discusses how disparities in access to digital tools and participation in online learning influenced how some benefited from the student-centred learning approaches adopted at this time. The emergence of the pandemic prompted fundamental shifts in teaching and learning, as South African universities had to implement several interventions to enable emergency remote education. Some of these changes helped pivot aspects of higher education from teacher-centred to more student-centred, as students were thrust into the driving seats of their educational journeys. Some of these student-centred approaches included increased asynchronous online lectures, flexibility in assessments, and structuring of course content to encourage more student-directed engagement with the resources. Thus the question the study seeks to answer is: To what extent did the digital divide influence the experiences of journalism students with emergency remote student-centred learning adopted due to the COVID-19 pandemic?

### Literature review

Student-centred learning is an approach to teaching and learning that emphasises the “importance of students’ past experiences, exploring individual needs and interests, promoting active participation, stimulating higher-order thinking, and encouraging life-long learning” (Hirumi 2002, p. 497). It is an educational approach rooted in constructivism theories that move away from the unidirectional orientation of teacher-centred education and assumes that for meaningful learning to occur, students must be given a chance to actively construct knowledge themselves (Neumann 2013). At the heart of this educational approach is a move towards centring students in the learning process, as opposed to centring teachers. O’Neill and McMahon (2005, p. 27) describe this as a “shift in power from the expert teacher to the student learner, driven by a need for a change in the traditional environment”.

This approach to education, typically referred to as the Presentation - Practice - Production lesson, rejects the reliance on conventional teaching approaches, such as synchronised lectures, and summative assessments, such as end-of-year exams, where learning is an activity driven by the teacher (Criado 2013; Maftoon and Sarem 2015). Instead, it promotes student-driven inquiry, where the teacher’s role is to ask questions and provide guidance when required, and it employs self-assessment, where students evaluate their own learning (Garrett 2008). As an educational concept dating back as early as 1905 through the work of Hayward, student-centred learning is a well-developed framework with various models for ascertaining its application and applicability in different contexts (O’Sullivan 2004). For this study, we have considered the seven tenets outlined by Lea et al. (2003, p. 322):

1. the reliance on active rather than passive learning,
2. an emphasis on deep learning and understanding,
3. increased responsibility and accountability on the part of the student,
4. an increased sense of autonomy in the learner
5. an interdependence between teacher and learner,
6. mutual respect within the learner-teacher relationship,
7. and a reflexive approach to the teaching and learning process on the part of both teacher and learner.

Scholars attribute several educational benefits in adopting this approach, including an increased motivation on the part of students to learn, increased independence and responsibility on the part of the student, interdependence between students and educators, as well as an emphasis on deep and active learning and understanding (Garrett 2008; Lea et al. 2003). Further, technologically enabled remote education is often compatible with student-centred learning because it allows students flexibility and access to a wide range of resources for their inquiry. It can also occur anywhere, provided there is connectivity and a suitable device (Hodges et al. 2020).

While student-centred learning is lauded as an approach that fosters “meaning making, inquiry and authentic activity” (Garrett 2008, p. 14), there is recognition that it is best suited for contexts where students have resources to embark on their inquiry. These resources include educational texts, such as books and journals, infrastructure and equipment, and a conducive learning environment (Matsilele and Nkoala 2022). It is also best employed in contexts where the approach is adopted at all educational levels, including primary education, because of the considerable change in basic assumptions of this approach (De la Sablonnière et al. 2009, p. 631).

Western countries, such as the United States, Canada, and countries in the European Union, primarily are where this education paradigm is used due to access to resources. In their paper on student-centred learning in Kyrgyzstan De la Sablonnière et al. (2009) note that because of the strict and coercive social and political structure there, the teacher-as-expert view is deeply entrenched, and as such a method that promotes independent enquiry on the part of students is likely to create a sense of dissonance in their learning. Similarly, studies that explore student-centred learning approaches in the African context highlight inadequate education systems, poor pedagogical understanding among teachers and lack of technological infrastructure as factors that have hindered its effective adoption on the continent. One of the critical arguments made by Schweisfurth (2011), for instance, is that when an education system does not use digital tools, its effort to employ the student-centred learning approach will not succeed. These technologies are rich enough to house various resources and prompt students towards learning that they have negotiated and co-constructed (Soong 2008). The slow and disjointed adoption of technology in higher education institutions in Africa highlighted in Anyanwu and Iwuamadi’s (2015) work on student-centred higher education in Nigeria, finds that while the concept of student-centred learning is embraced in Nigerian tertiary institutions, there is extraordinarily little evidence of this being the case in practice. Meanwhile, Nykiel-Herbert (2004) suggests that the economic and social disparities in South Africa mean that student-centred learning can only benefit particular students.

Journalism studies is a field that draws on student-centred teaching approaches by encouraging students to engage in independent study based on real-life developments and undertake assessments that promote deep engagement and learning, mainly driven by students’ curiosity (Hume 2007). It is also a field of study that is highly technologically driven, as students must be trained to use the digital tools they will employ when they enter the profession. In South Africa, universities usually play an essential role in mitigating disparities in access by providing computer laboratories and other equipment for students who do not own these. However, the sudden move to wholly online and remote learning due to the COVID-19 pandemic in 2020 changed this. As such, the experiences of the journalism students discussed in this paper provide an interesting case study for considering how disparities in access to digital tools and participation in online learning influenced the extent to which some derived benefit from student-centred learning approaches. Of course, neither the notion of student-centred learning nor that of the digital divide are novel in and of themselves. However, considering how these two topics influenced each other amid the unusual context of the emergency remote education due to the COVID-19 pandemic provides valuable insights for assessing how the digital divide can hinder students from enjoying the educational benefits that are supposed to come with student-centred learning.

### Framework: the digital divide and the usage gap

The digital divide is a theory that describes the “division between people who have access and use of digital media and those who do not*”* (Van Dijk 2020, p. 2). Three perspectives have been used to consider this idea. The first is innovation, wherein the digital divide considers how people can accept the development of new technologies. The second is about inequality and assesses the degree to which opportunities exist for people to adopt and use information and communication technology. Finally, the third perspective examines how technology enables or excludes people from participating in society. Of particular relevance to this study is Van Dijk’s theory of the ‘usage gap’, which combines all three perspectives. Van Dijk defines the usage gap as:

a systematic use of the Internet for particular goals by people of higher social class (education, income and property) and status (social position and cultural resources) as compared to those of lower social class and status. The goals are advanced information, communication and education, work, business and capital-enhancing or career activities (higher social class) as opposed to simple information and communication (chatting or messaging), shopping and entertainment (lower social class) (Van Dijk 2020, p. 98)

Van Dijk’s view is that people’s social standing determines how much they can harness digital technologies to attain their goals. His central argument is that unequal access to digital technologies brings about unequal participation in society and results in the unequal attainment of goals such as education and income. In this study, this notion of the digital divide is used to explain the disparities in the experiences of students when it comes to their ability or inability to benefit from the student-centred learning education approaches that were adopted in journalism studies as a result of the move to the wholly online teaching and learning necessitated by the COVID-19 pandemic in 2020. The argument is that given the growing centrality of digital tools in South African higher education, primarily fast-tracked by the COVID-19 pandemic, tertiary educators must be mindful of the disparities in access and participation due to the digital divide. As such, they should consider the student-centred approaches adopted in online teaching and learning through the lens of the digital divide; otherwise, inequalities will be exacerbated in terms of which students benefit.

## Methodology

### Research context

This study was undertaken at the Cape Peninsula University of Technology (CPUT), a South African higher education institution that largely serves students of colour. CPUT is the largest public tertiary institution in a region in South Africa known as the Western Cape province, with over 30 thousand students. It is also the only university of technology (UoT) in this province, with the other three public higher education institutions here being research-intensive universities. The reason for choosing a UoT is because over and above the theoretical training in a specialised field, the orientation of the academic programmes at these institutions is geared at educating students towards particular professions. As such, hands-on training and innovative problem-solving are central to teaching and learning, and many courses are taught with practicals as a key component of the curriculum. Further, these types of higher education institutions are intended to “build and nurture the social mobility of a large (predominantly black) highly skilled labour force” (Ndlevu 2017, p. 16).

The journalism programme considered as the site of study in this paper already employed several student-centred learning approaches before the COVID-19 pandemic due to the nature of the discipline. These strategies, which included students working in groups, using project- and problem-based learning strategies that encourage autonomy and authentic learning experiences, and giving students some choice when it comes to assessments, leveraged digital tools to personalise and deepen the learning experience. Before the emergency remote learning that had to occur, these approaches were strongly supported by a conducive context on campus where institutional resources ensured more equitable access to the required technologies. However, when social distancing was enforced to deal with COVID-19, and students suddenly had to engage in wholly online ermoted learning, the employment of student-centred learning in journalism studies was affected.

The group of students had differing levels of access to the digital tools needed to undertake their studies, and the lecturers had to change their curricula from a blended learning approach that used both online and in-person learning contexts to a completely virtual one where education could only take place through a technological medium. Practical demonstrations could also not occur as they had done in the past due to restrictions that prevented students from gathering on campus for several months.

### Data collection

This is a qualitative inquiry that employed a case study as its approach. The data consisted of 113 vlogs created by second and third-year students from a university of technology over a month from June 1 to June 30, 2020. Vlogs are usually short videos people produce to articulate their reflections on an issue. They are reflective tools that prompt self-evaluation (Brott 2021). They are also an archiving mechanism that allows one to observe how their reflections have changed over time. Further, unlike journals, vlogs allow for immediate and in-the-moment capturing of one’s thoughts and insights, especially if they are unscripted, which was the case for this study.

Every week for the first four weeks of online learning in 2020, the second and third-year students had to create vlogs where they reflected on teaching and learning during the pandemic and lockdown to capture their reflection-in-action (Little 2010). The vlogs averaged a minute long to capture only the uppermost issues in students’ minds. The theme of the reflections was “COVID-19 and its impact on journalism and journalism education”. The recordings were transcribed, and the analysis was based on this.

### Data analysis

The overarching research question was: to what extent did the digital divide influence the experiences of journalism students with emergency remote student-centred learning adopted due to the COVID-19 pandemic? As such, an analysis was undertaken using Clarke and Braun’s (2012) thematic analysis. The ‘top-down’ approach was based on three of seven tenets outlined by Lea et al. (2003, p. 322). These were chosen because they were the most prominent in what students mentioned in their vlog reflections. Thus the themes discussed are.

1. Disparities in the extent to which students engaged in active rather than passive learning
2. Disparities in the extent to which students demonstrated increased responsibility and accountability
3. Disparities in the extent to which students exhibited a sense of autonomy

## Discussion

### Disparities in the extent to which students could engage in active rather than passive learning

One of the significant changes instituted in the journalism programme discussed in this study was the inclusion of LinkedIn Learning as a resource. On its website, LinkedIn learning describes itself as an American “online educational platform that helps you discover and develop business, technology-related, and creative skills through expert-led course videos.” (Linkedin 2019). Lecturers used LinkedIn Learning particularly for practical courses, such as audiovisual production, because the emergency nature of the move to wholly online learning meant that there were no pre-recorded lectures of a practical nature at the time, such as how to use certain types of equipment and software. The LinkedIn Learning courses assigned to students to complete were aimed at mitigating this.

Students were directed to content relevant to the journalism diploma qualification they were working towards, such as courses on audiovisual editing, media law and documentary production. However, because LinkedIn Learning has many other courses, students could also engage with educational content not prescribed by their lecturers but rather out of their own interest. These courses, which include public speaking, time management, and animation, allowed students to engage in self-directed learning and develop skills beyond the stipulated programme objectives. As one student explained it:

*“*We also use LinkedIn learning which is a great resource for learning new skills*”* (Third year, male, vlog 1).

The technology provided some with opportunities to learn skills that might not have been gained in the traditional classroom. In addition, the self-paced orientation of this resource meant that students could go through the material when it was most suitable for them. This type of resource diminished the role of the lecturer as the expert and instead put students in the driving seat of their own skills acquisition. Finally, it presented some students with choices, which is vital for encouraging students to engage in active learning (Benson 2013).

This active learning, however, was severely limited for some students.

*“*COVID-19 has basically changed university life. The integration from contact classes to online learning has been quite difficult, given the issue of access for the majority of learners that don’t have, so this brings up the issue of inequality*”* ( Third year, female, vlog 1).

This student’s vlog reflection highlighted students’ difficulties with changing from contact classes to online learning. Her difficulty was not participating because of a lack of resources to engage in the online environment. However, she did not make pronouncements on the difficulty of the actual process of learning online. Thus one cannot say definitively whether she deemed it advantageous to learning in an online context compared to in-person classes. It is worth highlighting that while student-centred learning in this context seems to have been positive, this experience is for those students with access. Those who did not could not ascertain whether the experience would be positive or negative because they were excluded.

*“*With a lockdown, I really haven’t been able to do a lot of work because firstly I didn’t have a Wi-Fi connection... It’s just stressful because a lot of the work has piled up*”* (Third year, female, vlog 1).

With the move to online learning, the campus and the classroom, which can sometimes act as the great equaliser when it comes to accessing resources such as equipment and the internet in unequal societies like South Africa, are removed from the equation. Those with the means go on to not only enjoy the benefits of having access but the benefits that come with the student-centred learning that this access to technology enables. On the other hand, those who do not have access suffer the consequences of not having the technology to do the work. However, they must navigate the stress of finding other means of engaging in the content when the only available means require access. They are the ones who find themselves overwhelmed by the amount of work to be done because part of online learning is a scaffolded approach to tasks such that one assessment feeds into the next. A student who comes on board late finds themselves having to catch up on tasks while navigating the consequences of not having access.

“I could not attend classes as my municipality faced blackouts during working hours. This meant I could only attend to my academics from around 9 pm until 7 am. Those are the hours when we usually have electricity” (Third year, female, vlog 2).

Lack of access to reliable electricity and the internet limited participation for students like this. Thus even though the resource was made available to them, these students were precluded from full participation, limiting their ability to engage in active learning to the same degree as those with no access issues. Writing about the consequence of disparities in access to digital tools, Van Dijk (2020, p. 69) notes that “Someone who possesses high-quality examples of all available technical resources will benefit more than someone who has only one inferior device, a slow connection and a basic Internet subscription.” That is indeed the case, as reflected in these students’ experiences. The inequitable access to internet connectivity resulted in disparities in the extent to which students could engage in active learning through an educational tool like LinkedIn Learning. For those with full access, LinkedIn Learning enhanced their ability to be active learners, even more so than what would have happened in a classroom context. Conversely, for those with limited access, the usage gap was widened because not only were they missing out on the content they were prescribed to study, they also missed out on the benefit of learning extra skills.

### Disparities in the extent to which students could demonstrate increased responsibility and accountability

Another critical tenant of student-centred learning is that assessments should be used as and for learning. “If we are to optimise our students’ opportunities to learn, we must allow them, when possible, to show us what they have learned in ways that are optimal for them” (Doyle 2008, p. 101). This means reconsidering how we assess and, more fundamentally, why we assess. One of the things that emerged in this study is that due to the digital divide, there were disparities in the extent to which students could demonstrate responsibility and accountability, particularly regarding assessments. Even though the technology enabled autonomy and choice for some students, external directives, such as institutional requirements around marks being submitted on a specific date, meant that some aspects remained teacher-centred.

“However, our assignments and deadlines remain the same” (Third year, male, vlog 1).

Remarks like this were common in students’ vlog reflections. They pointed out that while the learning content changed when using platforms like LinkedIn Learning, several learning outcomes remained unchanged. Another response from a student in a different year of study supports the above comment.

“It depends on you as an individual into keeping the data and momentum of waking up every morning to attend classes” (Second year, female, vlog 1).

These remarks bring to the fore the need to consider how emergency remote learning, which we argue in some respects facilitated student-centred learning, is still bound by some of the traditional teacher-centred learning aspects, such as predetermined outcomes that do not change when the context and content of the lecture change.

On the other hand, there were aspects where students remarked that even amid the fixed deadlines, and perhaps because of them, they developed a sense of responsibility to self, spurred on by the fact that they were still accountable to complete their tasks by a particular day. This reality brought with it the challenges of time and personal management. One student shared her experiences in the following way:

“It has been difficult to motivate myself to get up and attend classes in the morning. And then self-learning has been a challenge as you must go through content by yourself; obviously, lecturers are there to help you when things are not clicking” (Second year, female, vlog 1).

This student makes a case around the challenges of self-study, especially for students from UoTs who are used to contact sessions and consistent engagement with their lecturers and peers. Here, the responsibility came through the fact that they could now determine for themselves when and how they worked due to the flexibility afforded and having a clear target to work towards because there was a fixed deadline. For example, one student explained it as follows:

“During this time, the pandemic has taught me to be punctual and to be self-motivated in order to succeed” (Third year, male, vlog 1).

For this student, the learning conditions created by the pandemic developed soft skills that they think would not have otherwise been developed. The suggestion is that the change in how things were done and the sense of urgency compelled the skills to rise to the fore. This remark is notable because assessments are often used to ascertain whether specific skills have been developed and certain knowledge gained in academic contexts. In this case, the situation compelled a student to develop these skills.

Further, for some students, the deadlines provided order. The fixed deadlines, characteristic of the teacher-centred approach, were much-needed sources of structure they could use to hold themselves accountable. One student stated:

“It’s better now because we have a date [for when to submit work]” (Third year, female, vlog 1)

This perspective suggests that having an end goal helped them establish structure and something to work towards. Thus, the requirement for deadlines in and of itself was not a dreadful thing. However, to make it more student-centred and mitigate against the disparities that exist due to access, consideration should be given to deadlines based on the extent to which students have access to the resources they need. For students with access and conducive studying environments to engage with the online content without hurdles, submissions of tasks could occur earlier. For students who come on board much later, submissions of tasks would happen much later. In this respect, there would be greater accommodation in assessment specifications so they are tailor-made for the student. This does not seem to be something the current way of teaching and learning considers.

“COVID-19 has had a negative impact on me as a journalism student because it is a practical subject. I need to be out there. I need to be meeting people. I need to be interviewing people. But I cannot do that now. It is so hard. I need to stay at home”. (Third year, female, vlog 3)

“The biggest concern I have in such uncertain times is with regard to work placement. Work placement is an important aspect in journalism. And it is essentially what my whole time in journalism education has led to... We will be forced to go the entrepreneurial route” (Third year, female, vlog 2)

The impact of the pandemic’s changes has been felt by the journalism industry and students working in the industry. The students’ remarks point to the fact that what is happening in education feeds into what is happening in the profession and vice versa. In this instance, the digital divide had a minimum impact because all of the students were in the same boat—none of them could engage in any projects that simulated the real-life context of a working journalist because of the restrictions to stay at home.

### Disparities in the extent to which students could exhibit a sense of autonomy

The notion of learner autonomy is really at the heart of student-centred learning in how it promotes learning that is less dependent on teachers and more centred on the student. One of the aspects that the vlog reflections touched on was the role of the learning environment in this. Students reflected on how moving from the traditional lecture room to learning virtually at home affected them. These reflections exposed another site of disparities due to the digital divide.

A student shared her sentiments, noting that remote learning and self-study were a challenge for her:

“One of the challenges I have had to deal with is staying motivated and working daily” (Second year, female, vlog 1).

Virtual learning, self-study and maintaining a sustained period of motivation were challenges most students faced during remote learning. As this student noted, staying focused on their studies was not easy, as doing academic work at home required self-discipline. In addition, virtual learning required personal motivation from the student.

Some students, such as the one quoted below, found the change extremely conducive:

“All of my classes are attended from the comfort of my own home” (Third year, male, vlog 1).

The home environment provided comfort for this student and was more convenient than the traditional classroom setup. Autonomy comes when students can learn in settings other than traditional classrooms, including their homes. While there are other forms of learning that students can undertake in contexts outside of their classrooms, it seems like, for this group, the fact that the delivery of lectures was occurring while there were at home was something that fostered a sense of autonomy. Before the COVID-19 pandemic, it was uncommon for students in UoTs in South Africa, particularly at the undergraduate level, to attend online classes on such a prolonged basis. This change created a sense of autonomy for students whose homes provided comfortable and conducive learning spaces.

For some students, though, learning at home was not easy:

“I have been hit hard by the pandemic because I cannot go to campus anymore now, meaning I have to study at home. Mind you the environment is not conducive for a student at all. So now I have to travel to meet with other colleagues to bounce off ideas. So, things have changed” (Third year, female, vlog 2).

Van Dijk (2020, p. 92) confirms this when he argues that “[u]sage of digital media is affected by the occasion, the obligation, the available time and the necessary effort expended. It depends on the *tasks* people have and the *contexts* in which they are living.” Students whose home context was optimal for individual study benefited, while those whose home context came with many inconveniences were disadvantaged. This disparity points to a need to provide students with options to undertake their studies in different environments if they can demonstrate the autonomy needed when engaged in student-centred learning. For some, it will include being able to stay at home. For others, it must be the campus context where resources not necessarily accessible at home exist.

“The COVID-19 pandemic has taught me that I need to be independent where I am not in class anymore or on campus to ask questions or ask one of my classmates. So, I am very independent. Also, I have to learn a lot of things. I have to Google a lot of things” (Third year, female, vlog 1).

Interestingly, the student equated independence with the classroom context firstly and secondly with not interacting with their peers. This remark suggests that the classroom context, in this view, creates a sense of dependence, where students look to the content delivered in that context to understand the concepts they are learning. Further, it seems that communication with peers in the classroom is where students deem peer-to-peer engagement to be most evident. Johnson et al. (2013) term this collaborative learning, which is learning that promotes group functioning. It thus enriches the learning experience by building students’ knowledge and enhancing their social skills. In this case, removing the peers from interacting in the classroom reduced communication outside the classroom.

“Online classes could never really replace in-person classes. First of all, you are not really motivated, and the environment is not really conducive. They are crying babies everywhere” (Third year, female, vlog 1).

Unlike the previous student, this idea of studying at home was more of trying to learn amid a chaotic domestic environment. Further, some students could not be autonomous because autonomy requires adequate resources anda conducive context. While they would have liked to drive their learning process, the lack of access to the essential resources needed to do that in this context meant that the education journey was difficult for them to navigate. Again, this difficulty is not at the level of the content but at the basic level of access, something that is all too common in this context where factors such as the digital divide mean that some have and others do not.

“Education for me isn’t as easy as it was supposed to be. I have to find software to edit. I have to struggle to find equipment to edit. I have to use cell phones for video” (Third year, female, vlog 1).

From this perspective, this student suggests that finding new ways of doing their tasks was a welcome challenge; for others, it felt like another hurdle to overcome in an already taxing course and learning climate. For students like this, the technological resources provided by the university would have improved the quality of their work because the resources they had access to were inadequate. Thus, while student-centred learning fosters independence and students’ ability to try and find things for themselves, this ability is closely tied to students looking for these things because of access. The contexts of this South African university of technology expose some reasons the student-centred learning framework might be viewed as a privilege of Western societies, where there is universal access to internet connectivity and the necessary technology.

“The move to online learning would have been more difficult if journalism students were not keeping up with the times” (Third year, female, vlog 1).

The student quoted above suggests that the nature of the journalism course is such that students are used to working independently, at their own pace on assessments, and remotely. Therefore, these changes would not have as significant an impact as would be the case in courses with summative assessments such as exams. In addition, the nature of journalism education, where a student is given a brief to interpret, means that some of the features of the student-centred learning experienced during the COVID-19 pandemic were already a way of life for some students.

Another student’s reflections on active learning were as follows:

“The unique thing about my course is that it focuses on unfolding events. Even though I am quarantined, I am able to further my studies as a budding journalist” (Second year, male, vlog 1).

Unlike many fields that required the normal functioning of the economy, for journalists and journalism students, COVID-19 presented new ways of learning through active observation of global developments around the pandemic. This student supports this notion by pointing out that the pandemic provided him with a learning opportunity as a budding journalist.

## Conclusion

When the World Health Organisation declared COVID-19 a global pandemic in 2020, universities worldwide underwent unanticipated changes in how they operated and delivered the academic programme. Chief among these changes was a wide-scale adoption of blended learning pedagogies. From the discussion above, the overall conclusion is that the digital divide influenced the experiences of journalism students with emergency remote student-centred learning to a large extent. The divide exacerbated disparities in how students could engage in active rather than passive education, in the extent to which students could demonstrate increased responsibility and accountability, and the degree to which students could exhibit a sense of autonomy. Those with physical access and who could participate meaningfully online derived even more benefit from the student-centred approaches employed during remote teaching and learning than when these approaches are used in blended or face-to-face education. On the other hand, those with limited access were disadvantaged further because not only could they not engage in learning, but they also could not participate in enriching activities, such as learning new skills through LinkedIn Learning, being able to study in a comfortable and homely environment and being able to make choices in terms of their assessments. This suggests that when student-centred learning approaches are used in the context of wholly online learning, the resulting disparities can have dire consequences for those excluded from meaningful engagement.

While some of the tenants of student-centred learning, as conceptualised in Western contexts, like the United States and Europe, are deemed to be positive in the vlog reflections of the second and third-year students featured in this study, there are nuances to them because of the disparities in technological access and participation that occur in the South African context due to the digital divide. One of the things that the prevailing conceptualisation of student-centred learning seems to take for granted is that students have a choice in their learning. Choice in learning is desirable for students but can hinder students without the means to choose due to limitations in access to learning resources. Crosby and Harden (2000) think of student-centred learning as what students do to achieve this rather than what the teacher does. There is also an emphasis on the power shift. The frameworks do not appear to consider some environments and situations where the choice is there for students, but students are unable to exercise it due to a lack of access to resources or a conducive environment as a result of the disparities that exist.

The current models and framework around student-centred learning focus primarily on knowledge acquisition and the processes used when students learn. This case study shows that access and context are also components to consider because, in a place like South Africa, where there are disparities in where students can and cannot learn, this becomes an important aspect to consider. The other element we see is that access to digital tools determines how desirable student-centred learning is. Where students do not have resources, it is more desirable that teacher-centred learning is employed because, at least then, students have access to resources and can acquire some knowledge. A student-centred approach will work when students have access to resources because they can then embark on inquiries themselves and not rely on the teacher directing them.

Thus, for example, one of the tenets of student-centred learning is student autonomy. Students need access to resources; otherwise, they cannot undertake the independent inquiry envisaged by affording them this autonomy. Similarly, a student in an environment that does not enable them to engage with learning may be presented with a choice in theory, but they cannot choose. In a context like South Africa, with education systems that are held back by disparities in access to resources and differences in learning environments, the conceptualisation of student-centred learning in online contexts should incorporate these elements; otherwise, it may always be assumed that factors standing in the way of greater adoption of student-centred learning are teachers who are beholden to teacher-centred learning due to assorted reasons. As higher education institutions are emerging from the worst of the pandemic, they are grappling with which aspects of some of the emergency pedagogies to discard and which to retain. The issues highlighted in this study demonstrate that as desirable as student-centred learning is for fields like journalism education, contextual issues, such as access to resources and a conducive environment, warrant educators to consider how to mitigate against disparaties that arise in unequal social contexts, like South Africa.

## Data Availability

The data for this study can be found on the following https://doi.org/10.6084/m9.figshare.17020028
